# Prenatal exposure to consumer product chemical mixtures and size for gestational age at delivery

**DOI:** 10.1186/s12940-021-00724-z

**Published:** 2021-06-10

**Authors:** P. A. Bommarito, B. M. Welch, A. P. Keil, G. P. Baker, D. E. Cantonwine, T. F. McElrath, K. K. Ferguson

**Affiliations:** 1 Epidemiology Branch, Division of Intramural Research, National Institute of Environmental Health Sciences, 111 T.W. Alexander Drive, Durham, NC 27709 USA; 2 Department of Epidemiology, Gillings School of Global Public Health, University of North Carolina at Chapel Hill, 135 Dauer Drive, Chapel Hill, NC 27599 USA; 3 NSF International, 789 N. Dixboro Road, Ann Arbor, MI 48105 USA; 4 Division of Maternal-Fetal Medicine, Brigham and Women’s Hospital, Harvard Medical School, 75 Francis Street, Boston, MA 02115 USA

**Keywords:** Organophosphate esters, Phthalates, Phenols, Chemical mixtures, Small-for-gestational age, Large-for-gestational age

## Abstract

**Background:**

While fetal growth is a tightly regulated process, it is sensitive to environmental exposures that occur during pregnancy. Many commonly used consumer products contain chemicals that can disturb processes underlying fetal growth. However, mixtures of these chemicals have been minimally examined. We investigated associations between prenatal exposure to 33 consumer product chemicals (nine organophosphate ester flame retardant [OPE] metabolites, 12 phthalate metabolites, and 12 phenols) and the odds of small- or large-for-gestational age (SGA and LGA) births.

**Methods:**

This case-control study was comprised of SGA (*N* = 31), LGA (*N* = 28), and appropriate for gestational age control (*N* = 31) births selected from the larger LIFECODES cohort. Biomarkers of exposure to consumer product chemicals were quantified in maternal urine collected from up to three study visits during pregnancy. In a single-pollutant approach, odds ratios (OR) and 95% confidence intervals (CI) of SGA and LGA associated with an interquartile range (IQR)-increase in exposure biomarkers were estimated using multinomial logistic regression. In a multi-pollutant approach, quantile g-computation was used to jointly estimate the OR (95% CI) of SGA and LGA per simultaneous one quartile-change in all biomarkers belonging to each chemical class.

**Results:**

Among the 33 biomarkers analyzed, 20 were detected in at least 50% of the participants. After adjusting for potential confounders, we observed reduced odds of LGA in association with higher urinary concentrations of several exposure biomarkers. For example, an IQR-increase in the OPE metabolite, diphenyl phosphate, was associated with lower odds of LGA (OR: 0.40 [95% CI: 0.18, 0.87]). Using quantile g-computation, we estimated lower odds of an LGA birth for higher OPE metabolite concentrations (OR: 0.49 [95% CI: 0.27, 0.89]) and phthalate metabolite concentrations (OR: 0.23 [95% CI: 0.07, 0.73]). Associations between consumer product chemicals and SGA were largely null.

**Conclusions:**

Joint exposure to OPEs and phthalates was associated with lower odds of delivering LGA. Associations with LGA could indicate a specific impact of these exposures on the high end of the birth weight spectrum. Future work to understand this nuance in the associations between consumer product chemical mixtures and fetal growth is warranted.

**Supplementary Information:**

The online version contains supplementary material available at 10.1186/s12940-021-00724-z.

## Introduction

Fetal growth is a tightly regulated process controlled by maternal and paternal genetics, maternal nutrition, metabolic function, endocrine activity, and epigenetics [1]. Deviations from average fetal growth increase the risk of adverse health outcomes later in life. For example, being born excessively small, often defined as small-for-gestational age (SGA), is a risk factor for infant mortality, altered childhood growth, neurodevelopmental disorders, and lower intellectual performance [2, 3]. At the other end of the growth spectrum, infants diagnosed as large-for-gestational age (LGA) are at increased risk of developing a range of cardiometabolic outcomes, such as high blood pressure, diabetes, and obesity [4–6]. Risk of adverse outcomes is most pronounced at the extremes of fetal growth, although even subtle changes in growth may be associated with adverse outcomes [7].

Fetal growth processes are sensitive to environmental exposures during pregnancy [8]. Among the chemicals to which pregnant women are exposed, there has been growing interest in a number of synthetic chemical classes found ubiquitously in consumer products. Such chemicals include organophosphate esters (OPE), which may be used in consumer products as either flame retardants or plasticizers, as well as phthalates and phenols. These chemicals, or their metabolites, are commonly detected in the urine of pregnant women [9–11]. Previous studies have linked the use of consumer products, such as cosmetics or household cleaners, to higher exposure to OPEs [9, 12], phthalates [11, 13], and phenols [10] in pregnant women. Exposures from consumer products likely occur through dermal or inhalation pathways and represent a substantial proportion of exposure to many of these chemicals [14, 15]. However, due to the ubiquitous use of these compounds in consumer products, they contaminate environmental media such as food and drinking water. Therefore, the ingestion pathway also contributes to internal levels of these compounds [15, 16].

Importantly, these consumer product chemicals are suspected to impact processes critical to the regulation of fetal growth, possibly through endocrine disruption or impacts on maternal oxidative stress and inflammation [1]. For example, prenatal phthalate exposure has been previously shown to promote maternal oxidative stress [17, 18], while phenol exposure has been associated with maternal inflammatory markers [19]. Many consumer product chemicals have also been demonstrated to have endocrine disrupting properties [20, 21].

Nevertheless, the literature linking consumer product chemicals with altered fetal growth is highly inconsistent [1]. These inconsistencies may be the result of differences in study design [22, 23], timing and frequency of exposure assessment [24–26], and the metrics used to monitor and define fetal growth outcomes [27–29]. In addition to these considerations, pregnant women are exposed to highly complex mixtures of consumer product chemicals [30, 31]. In general, the effects of chemical mixtures on fetal growth have not been thoroughly explored. Recent studies have examined mixtures of persistent organic pollutants [32], and trace metals [33, 34] in relation to fetal growth parameters. Attempts have also been made to estimate the maternal exposome, including chemical and non-chemical exposures, and its effects on birth weight [35]. Yet, the effects of consumer product chemical mixtures are understudied and may be of greater interest as these exposures may be intervenable upon through behavioral changes or regulatory measures, which may reduce or eliminate their use in consumer products [36, 37]. Within the mixture of consumer product chemicals, individual compounds may act antagonistically or synergistically with one another. However, it is important to note that consumer behavioral changes or regulatory limitations on consumer product chemicals will likely affect exposure to multiple chemicals simultaneously. Therefore, the joint effects of consumer product chemicals on fetal growth should be considered.

In the present study, we used a case-control population nested within an ongoing prospective birth cohort to investigate the association between consumer product chemicals and the odds of SGA and LGA births. Using urine samples collected at up to three study visits during pregnancy, we measured the concentrations of 33 biomarkers that indicate exposure to chemicals found in consumer products, including OPEs, phthalates, and phenols. We investigated single-pollutant associations with the odds of SGA and LGA births, as well as the joint effect of compounds within each individual chemical class using quantile g-computation.

## Methods

### Study population

We included a subset of participants from the larger LIFECODES study, an ongoing prospective pregnancy cohort that began in 2006 at Brigham and Women’s Hospital (BWH) in Boston, MA. To be eligible for the study, women must be at least 18 years of age, seek prenatal care before 15 weeks gestation, and intend on delivering at BWH. At the first study visit (median 11 weeks gestation), participants provide informed consent, complete detailed questionnaires about demographics and medical history, and provide urine samples. Gestational age is estimated according to the American College of Obstetrics and Gynecologists (ACOG) using the last menstrual period with verification by ultrasound measures [38]. Participants subsequently attend two additional study visits (median 26 and 35 weeks gestation) at which additional urine samples are collected. All samples are frozen and stored at − 80 °C until analysis. This research was approved by the Institutional Review Board at BWH.

The present study is a pilot study that was conducted in a nested case-control population which was designed to examine associations between prenatal consumer product chemical exposures, inflammation biomarkers, and fetal growth [39]. Birth weight percentiles for gestational age were calculated according to guidelines established by Oken et al. [7]. These percentiles were first used to randomly select pregnancies that result in SGA (*n* = 30) births. Subsequently, appropriate for gestational age (AGA; *n* = 30) and LGA (*n* = 30) births were selected. SGA was defined as a birth weight-for-gestational age < 10th percentile; LGA as > 90th percentile; and AGA between 10th – 90th percentiles. AGA and LGA births were matched to SGA births in a 1:1:1 ratio with frequency matching based on the following criteria: maternal age (± 5 years), maternal race (white/Black/other), maternal pre-pregnancy body mass index ([BMI], ± 5 kg/m^2^), and gestational age at delivery (± 2 weeks). Pregnancies included in this study occurred between 2010 and 2017 and in order to be eligible for selection into the case-control study, women were required to have singleton pregnancies. Additionally, women were not eligible for selection if they had pre-existing diabetes or if their pregnancy had congenital malformations. Following participant selection and laboratory analyses for this study, we identified a minor error in the coding of birthweight percentiles. When corrected, a total of three participants (3% of the total sample) were recategorized. Specifically, two participants previously believed to be LGA were recategorized as AGA, and one participant previously believed to be AGA was recategorized as SGA. Therefore, the revised distribution of cases and controls is: 31 SGA, 31 AGA, and 28 LGA births. A flow-through diagram detailing selection from the parent LIFECODES study can be found in Additional File 1: Supplemental Fig. 1.

### Urinary quantification of exposure biomarkers

Urine samples from each study visit were sent to NSF International (Ann Arbor, MI) for analysis of exposure biomarkers. Prior to shipment, samples were randomized across batches in order to reduce impact of batch effects and analysts were blinded to sample characteristics. Exposure to a total of 33 consumer product chemicals was assessed using panels of OPE metabolites (k = 9), phthalate metabolites (k = 12), and phenolic compounds (k = 12). A list of all analytes, abbreviations, and their corresponding parent compounds can be found in Additional File 1: Supplemental Table 1.

Quantification of the OPE metabolites was performed in previously unthawed urine samples using isotope dilution–liquid chromatography–tandem mass spectrometry (ID–LC–MS/MS). The method was developed to simulate the Centers for Disease Control and Prevention analytical method [40] and was validated in accordance with Food and Drug Administration recommendations [41]. Additional details for the method of OPE quantification are provided in Additional File 2: Appendix 1. The quantification of both phthalate metabolites and phenols has been previously described in detail [42, 43]. For the analysis of phthalate metabolites, conjugated species were hydrolyzed using β-glucuronidase/E. coli K-12. Conjugated phenol species were also hydrolyzed prior to analysis using β-glucuronidase/sulfatase. After hydrolysis, both phthalate metabolites and phenols were analyzed using ID-LC-MS/MS. All analyses were carried out using a Thermo Scientific Transcend TXII Turbulent Flow system interfaced with Thermo Scientific Quantiva triple quadrupole mass spectrometer.

The limit of detection (LOD) and proportion of samples below the LOD for each exposure biomarker are included in Additional File 1: Supplemental Table 1. For biomarker concentrations that were below the limit of detection, machine-read values were used in this analysis [44]. When reported values were less than zero or blank, the value was imputed as LOD/√2 [45]. Only analytes with at least 50% detection across all study visits were included in further analysis. For these included biomarkers, over 96% of all samples had machine-read values available. The effect of urinary dilution on biomarker concentrations was accounted for using specific gravity (SG), which was measured using a digital handheld refractometer (AtagoCo., Ltd., Tokyo, Japan). Concentrations of urinary biomarkers were corrected for urinary dilution using the following formula: CP_SG_ = CP (1.016–1)/(SG_i_ – 1), where CP_SG_ is the SG-corrected measurement, CP is the measured concentration, 1.016 is the median SG in the study population, and SG_i_ is each individual’s urinary SG [46]. An individual’s average exposure to each chemical during pregnancy was estimated by taking the geometric mean of the SG-corrected biomarker concentrations measured at up to three study visits.

### Statistical analysis: single-pollutant approach

Statistical analysis was performed using SAS version 9.4 (SAS Institute; Cary, NC) and R version 3.6.3 [47]. The distributions of demographic factors were examined by calculating the median (interquartile range [IQR]) or n (%) according to case status. The median (IQR) of the average exposure biomarker concentrations were also calculated for study participants in the overall cohort and according to case status. Levels of exposure biomarkers in cases and controls were contrasted using Kruskal-Wallis tests. In addition, to examine how exposure varied by demographic characteristics, we calculated the median concentrations of each biomarker by key variables (i.e., age, BMI, race, etc.) and tested for differences in the median using Kruskal-Wallis tests. We quantified bivariate associations between biomarkers using a Spearman correlation matrix. Lastly, to estimate the stability of these biomarkers across study visits, intraclass correlation coefficients (ICC) and 95% confidence intervals (CI) were calculated using linear mixed effect models with random intercepts for each participant using the %ICC9 macro in SAS [48].

We used multinomial logistic regression models to estimate the odds ratios (OR) and 95% CI contrasting SGA and LGA with AGA for an IQR-increase in the concentration of each exposure biomarker. Exposure biomarkers were ln-transformed in order to reduce the impact of highly skewed biomarker distributions on model results. Adjusted models included the following variables that were used in frequency matching: age (years), pre-pregnancy BMI (kg/m^2^), and maternal race (white/Black/other). While we also matched based on gestational age at delivery, this factor was implicitly controlled for by defining growth outcomes using birth weight-for-gestational age z-scores, which are conditional on gestational age. Therefore, we did not include gestational age at delivery in our models. Based on a priori knowledge, we also assessed potential confounding by the following variables: maternal education (high school or less/some college or technical school/completed college or greater), maternal insurance (private/public), parity (nulliparous/parous), fetal sex (female/male), and calendar year of birth (years). These factors were retained in final models if their inclusion influenced estimates by > 10%. Given that there was sparse data across strata of maternal race and maternal education, analyses were replicated using collapsed categories (race: white/non-white and education: did not graduate college/graduated college). Results were unchanged (data not shown) and we maintained the original categorization to ensure comparability with other analyses on this cohort [39].

Next, we performed two sensitivity analyses. First, there is evidence that many endocrine disrupting compounds act in a sex-specific manner during development [49, 50]. Thus, we explored the potential for sex-specific effects by including product interaction terms for sex and each exposure biomarker [51]. From these models, we reported stratum-specific estimates as well as Wald *p*-values from the interaction terms. Second, we examined the impact of smoking and alcohol use on our findings. Given the small number of women who reported smoking cigarettes (*n* = 6 [7%]) or drinking alcohol (*n* = 6 [7%]) during pregnancy, we could not adjust for these covariates in our primary models and instead excluded individuals who reported their use.

### Statistical analysis: multi-pollutant approach

We used quantile g-computation to estimate the joint association between mixtures of consumer product chemical biomarkers and fetal growth. Quantile g-computation has been previously described in detail [52]. Briefly, this method estimates the change in the log odds of an outcome associated with a simultaneous one quantile increase in the exposure level of a pre-specified mixture. This approach makes no assumptions about the effect direction of any individual exposure. While we assume linearity and additivity of associations, we note that this approach allows extensions to accommodate non-linearity and non-additivity of individual exposure effects, as well as the effect of the mixture. Of relevance to this study, quantile g-computation has also been shown to reduce bias in small sample sizes compared to other methods that are commonly used to estimate joint effects, such as weighted quantile sum regression [52]. Ours is a novel application of this approach to a case-control study design. In cohort designs (and under appropriate causal identification assumptions), quantile g-computation estimates causal joint effects of exposure. Absent such assumptions, quantile g-computation estimates “joint associations” of exposure with outcomes, similar to other regression models. Because “joint associations” is not typical terminology, we refer to effect estimates as “joint effects.”

Under the assumption of linearity, quantile g-computation is conducted in three steps. First, the exposures of interest (i.e., the “mixture”) are transformed into quantiles. Second, a logistic model is fit regressing the outcome onto the set of quantized exposures and covariates. Third, the joint, or overall mixture effect is defined as the sum of regression coefficients for the exposures within the mixture. To quantify effects of individual exposures, weights calculated from the regression coefficient for each quantized exposure, which describe the proportion of negative or positive partial effects related to each individual exposure. In this analysis, we defined three mixtures based on belonging to a specific chemical class (i.e., OPEs, phthalates, and phenols) and estimated the OR (95% CI) of SGA or LGA associated with a one quartile increase in all exposures within a mixture. Given the small sample size and the lack of strong correlations between chemicals in different classes, we did not co-adjust our models for other chemical classes. In contrast to the single-pollutant models, quantile g-computation was carried out using two logistic models contrasting the odds of SGA or LGA to the odds of AGA (referent in both models). All models were constructed controlling for the same covariates as in our single-pollutant models. Quantile g-computation was carried out using the ‘qgcomp’ package version 2.3.0 [53].

## Results

Demographic characteristics for participants in this study are shown in Table 1. Overall, mothers with SGA, AGA, and LGA births were similar, as expected due to matching. Women in this study had a median age of approximately 33 years and a pre-pregnancy BMI in the normal/overweight range. They were predominantly white, with a college education and access to private health insurance. In addition, parity was similar between cases and controls. Few women reported using cigarettes or consuming alcohol during pregnancy. The distribution of fetal sex was also similar between cases and controls. Births classified as SGA had a median birth weight of 2.3 kg, while the median birth weights for AGA and LGA were 3.2 kg and 4.2 kg, respectively. Women selected into this study are similar to those in the parent LIFECODES cohort (Additional File 1: Supplemental Table 2).

**Table 1:** Demographic characteristics of study participants by case status

	Median (25th, 75th percentile) or n (%)		
	SGA (***n*** = 31)	AGA (*n* = 31)	LGA (*n* = 28)
**Matched Variables**			
Maternal Age (years)	33.6 (26.9, 37.3)	32.4 (30.4, 37.5)	34.5 (30.4, 37.1)
Pre-pregnancy BMI (kg/m^2^)	23.0 (20.2, 27.9)	23.0 (22.3, 27.4)	25.7 (22.1, 27.9)
Race/Ethnicity			
White	19 (61)	18 (58)	17 (61)
Black	6 (19)	6 (19)	6 (21)
Other^1^	6 (19)	7 (23)	5 (18)
Gestational Age (weeks)	37.9 (37.1, 38.6)	39.0 (38.3, 39.6)	39.1 (38.8, 39.8)
**Non-matched Variables**			
Birth weight (kg)	2.3 (2.2, 2.4)	3.2 (3.0, 3.4)	4.2 (4.1, 4.3)
Birth weight percentile (%)	3.0 (1.0, 4.0)	35.0 (23.0, 46.0)	96.0 (93.0, 97.5)
Maternal Insurance			
Private	22 (71)	24 (77)	20 (71)
Public	9 (29)	7 (23)	8 (29)
Maternal Education			
≤ High School	3 (10)	3 (10)	1 (4)
Some college or technical school	8 (26)	8 (26)	6 (21)
≥ College graduate	20 (65)	20 (65)	21 (75)
Parity			
Nulliparous	7 (23)	10 (32)	7 (25)
Parous	24 (77)	21 (68)	21 (75)
Smoking During Pregnancy			
No	27 (87)	29 (94)	28 (100)
Yes	4 (13)	2 (6)	0 (0)
Alcohol Use During Pregnancy			
No	29 (94)	28 (90)	27 (96)
Yes	2 (6)	3 (10)	1 (4)
Fetal Sex			
Female	19 (61)	18 (58)	13 (46)
Male	12 (39)	13 (42)	15 (54)

Abbreviations: IQR = interquartile range; BMI = body mass index

^1^The “Other” race/ethnicity category is a condensation of larger categories with insufficient sample size for disaggregation (e.g., Hispanic/Latino ethnicity, South Asian, East Asian, Native American, multiracial)

Of the 33 analytes measured, 20 were detected in at least 50% of the samples at each study visit: two OPE metabolites, 11 phthalate metabolites, and seven phenolic compounds (Additional File 1: Supplemental Table 1). Within study visits, most participants had exposure biomarker measurements available at all three study visits, although some only provided two samples (*n* = 5) and one individual provided a single sample. Overall, samples were more likely to be missing at the last study visit (*n* = 89 at visit 1, *n* = 89 at visit 2, and *n* = 85 at visit 3) and were more likely to be missing in SGA (*n* = 4) cases compared to LGA (*n* = 2) or controls (*n* = 0). There were several differences in the average exposure biomarker concentrations between cases and controls in this study (Table 2). Urinary concentrations of mono-ethyl phthalate (MEP) and methyl paraben (MPB) were highest in mothers who went on to have AGA births and lowest in those who had LGA births. Similar trends were also noted for diphenyl phosphate (DPhP) and propyl paraben (PPB). Median concentrations and IQR of the uncorrected average exposure biomarkers and detection frequencies according to case status are reported in Additional File 1: Supplemental Table 3.

**Table 2:** Median (25th, 75th percentile) SG-adjusted average urinary concentrations of exposure biomarkers (ng/mL) in the overall study population and according to case status

Chemical Class	Urinary Analyte (ng/mL)		Median (25th, 75th)			
		Overall	SGA	AGA	LGA	*p*^**1**^
		(***n*** = 90)	(*n* = 31)	(***n*** = 31)	(*n* = 28)	
OPEs	BDCPP	0.67 (0.40, 1.09)	0.84 (0.33, 1.35)	0.79 (0.44, 1.12)	0.51 (0.35, 0.78)	0.14
	DPhP	0.74 (0.52, 1.22)	0.72 (0.47, 1.60)	0.82 (0.70, 1.34)	0.60 (0.45, 0.89)	0.07
Phthalates	MEP	42.9 (14.5, 128)	41.1 (8.48, 188)	65.1 (28.7, 133)	26.4 (11.7, 60.0)	0.03
	MBP	9.51 (6.81, 12.9)	9.99 (6.27, 17.9)	10.5 (7.14, 16.8)	8.36 (5.92, 10.5)	0.16
	MBzP	3.27 (1.86, 6.85)	3.57 (1.89, 7.55)	2.68 (1.55, 5.88)	3.39 (1.88, 6.78)	0.78
	MiBP	5.69 (4.04, 8.84)	5.73 (4.26, 9.24)	6.18 (3.78, 9.95)	5.47 (4.03, 7.08)	0.30
	MECPP	9.17 (5.81, 15.9)	11.1 (5.87, 18.5)	9.37 (5.81, 17.7)	8.52 (4.76, 15.5)	0.56
	MEHHP	6.31 (4.45, 10.9)	7.60 (5.26, 10.9)	5.30 (4.29, 11.1)	5.98 (3.92, 10.4)	0.24
	MEOHP	4.44 (3.09, 7.88)	5.32 (3.26, 7.88)	3.77 (3.21, 8.02)	4.18 (2.58, 7.22)	0.58
	MEHP	1.89 (1.16, 2.90)	2.28 (1.34, 3.09)	1.76 (1.02, 3.26)	1.68 (1.08, 2.59)	0.38
	MCPP	2.24 (1.28, 4.50)	2.13 (1.13, 5.97)	2.47 (1.64, 5.23)	2.04 (1.08, 3.43)	0.42
	MCOP	2.02 (1.09, 5.66)	3.45 (1.09, 5.25)	2.03 (1.17, 6.79)	1.73 (0.71, 4.82)	0.42
	MCNP	0.30 (0.21, 0.46)	0.31 (0.23, 0.50)	0.31 (0.23, 0.48)	0.27 (0.17, 0.40)	0.36
Phenols	2,4-DCP	0.31 (0.21, 0.56)	0.31 (0.21, 0.60)	0.28 (0.21, 0.51)	0.37 (0.23, 0.57)	0.60
	2.5-DCP	0.62 (0.42, 1.36)	0.79 (0.42, 1.45)	0.59 (0.43, 1.47)	0.57 (0.32, 1.18)	0.54
	BP3	34.7 (12.3, 95.0)	45.6 (9.20, 95.0)	26.7 (13.7, 89.4)	44.0 (13.2, 128)	0.81
	BPA	0.63 (0.48, 0.92)	0.59 (0.48, 0.99)	0.62 (0.42, 0.90)	0.74 (0.50, 1.01)	0.54
	MPB	105 (37.3, 188)	125 (56.8, 204)	125 (76.4, 219)	37.0 (24.9, 170)	0.01
	PPB	19.0 (7.07, 40.0)	18.6 (7.07, 50.7)	23.8 (12.1, 52.5)	10.7 (4.89, 29.6)	0.05
	TCS	6.30 (2.09, 33.3)	4.24 (1.44, 44.1)	5.57 (1.61, 15.7)	12.3 (3.32, 52.4)	0.13

^1^*p-value*: Kruskal-Wallis Test

Exposure biomarker concentrations differed by several demographic characteristics (Additional File 1: Supplemental Table 4). For example, urinary concentrations of several phthalate metabolites and all measured phenols varied across maternal race categories, where most analytes were highest among Black women or women of other race/ethnicity, and lowest among white women. Levels of OPE metabolites did not vary across many demographic characteristics, although bis (1,3-dichloro-2-propyl) phosphate (BDCPP) was higher among participants reporting the use of public compared to private health insurance and was marginally higher among individuals with overweight or obese BMI compared to those with a normal/underweight BMI.

**Fig. 1:**
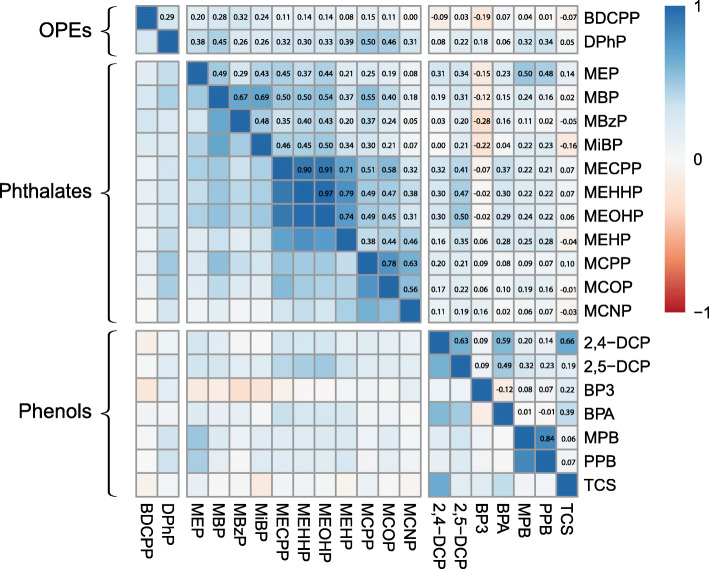
Spearman correlation coefficients for average exposure biomarker concentrations

Overall, the exposure biomarkers were low-to-moderately correlated with one another (Fig. 1). The strongest positive correlations were observed among phthalate metabolites, particularly among di-2-ethylhexyl phthalate (DEHP) metabolites (ranging from spearman’s rho [r] = 0.71–0.97). In addition, MPB and PPB were highly correlated (r = 0.84), likely reflecting similar usage in consumer products. In line with previous studies using repeated measures of these biomarkers, there was poor-to-fair stability for all analytes measured (0.25 ≤ ICC ≤ 0.74; Additional File 1: Supplemental Table 5) [10, 11, 54].

After adjusting for age, pre-pregnancy BMI, maternal race, maternal education, and fetal sex, we observed inverse associations between several biomarkers and LGA. Specifically, an IQR-increase in average urinary concentrations of DPhP (OR: 0.40 [95% CI: 0.18, 0.87]), MEP (OR: 0.33 [95% CI: 0.14, 0.78]), MPB (OR: 0.25 [95% CI: 0.10, 0.63]), and PPB (OR: 0.34 [95% CI: 0.14, 0.78]) were associated with reduced odds of LGA (Table 3). Crude results were similar (Additional File 1: Supplemental Table 6). Associations between exposure biomarkers and SGA did not meaningfully deviate from the null.

**Table 3:** Adjusted^1^ OR (95% CI) of SGA and LGA associated with an IQR-increase in average urinary exposure biomarker concentrations

Chemical Class	Urinary Analyte	SGA	LGA
		aOR^**1**^ (95% CI)	aOR^**1**^ (95% CI)
OPEs	BDCPP	1.12 (0.57, 2.18)	0.56 (0.27, 1.16)
	DPhP	1.01 (0.55, 1.87)	0.40 (0.18, 0.87)*
Phthalates	MEP	0.56 (0.27, 1.19)	0.33 (0.14, 0.78)*
	MBP	1.26 (0.78, 2.06)	0.71 (0.41, 1.25)
	MBzP	1.37 (0.67, 2.79)	1.00 (0.46, 2.17)
	MiBP	1.14 (0.60, 2.19)	0.54 (0.25, 1.16)
	MECPP	1.08 (0.49, 2.36)	0.64 (0.28, 1.47)
	MEHHP	1.41 (0.70, 2.86)	0.83 (0.39, 1.77)
	MEOHP	1.28 (0.59, 2.78)	0.81 (0.36, 1.83)
	MEHP	1.18 (0.60, 2.31)	0.74 (0.36, 1.52)
	MCPP	0.83 (0.42, 1.64)	0.56 (0.27, 1.18)
	MCOP	0.87 (0.38, 1.99)	0.57 (0.24, 1.36)
	MCNP	0.89 (0.47, 1.68)	0.52 (0.25, 1.06)
Phenols	2,4-DCP	1.17 (0.54, 2.53)	1.27 (0.58, 2.81)
	2,5-DCP	0.98 (0.50, 1.89)	0.70 (0.34, 1.42)
	BP3	0.75 (0.36, 1.54)	0.79 (0.38, 1.65)
	BPA	0.97 (0.55, 1.72)	1.42 (0.78, 2.60)
	MPB	0.91 (0.42, 1.95)	0.25 (0.10, 0.63)*
	PPB	0.80 (0.38, 1.71)	0.34 (0.14, 0.78)*
	TCS	1.09 (0.44, 2.69)	1.95 (0.80, 4.79)

Asterisks indicate *p* < 0.05

Abbreviations: IQR = interquartile range; aOR = adjusted odds ratio

^1^Models adjusted for age (years), pre-pregnancy BMI (kg/m^2^), maternal race (white/Black/other), maternal education (high school or less/some college or technical school/completed college or greater), and fetal sex (female/male)

Effect estimates were similar between strata of male and female infants, with the exception of several DEHP metabolites (i.e., mono-(2-ethyl-5-carboxypentyl) phthalate [MECPP], mono-(2-ethyl-5-hydroxyhexyl) phthalate [MEHHP], mono-(2-ethyl-5-oxohexyl) phthalate [MEOHP], and mono-2-ethylhexyl phthalate [MEHP]), which exhibited suggestive sex-specific trends with respect to SGA (Additional File 1: Supplemental Table 7). Effect estimates, though imprecise, were above the null among female infants and below the null for male infants for associations between all DEHP metabolites and SGA. For example, an IQR-increase in MEHHP was associated with an OR of 3.10 (95% CI: 1.04, 9.24) among female infants and an OR of 0.63 (95% CI: 0.22, 1.80) among male infants (Wald *p*-value = 0.04 for interaction). There was little evidence for heterogeneity by sex for other exposure biomarkers with respect to SGA births. Similarly, few differences were observed by sex for LGA births. However, we note that stratum-specific estimates tended to be highly imprecise due to the small study sample.

Lastly, we conducted a sensitivity analysis to examine potential confounding by cigarette and alcohol use during pregnancy. Effect estimates were not meaningfully different from primary results when individuals reporting the use of cigarettes and alcohol were excluded from the analysis (Additional File 1: Supplemental Table 8).

**Fig. 2:**
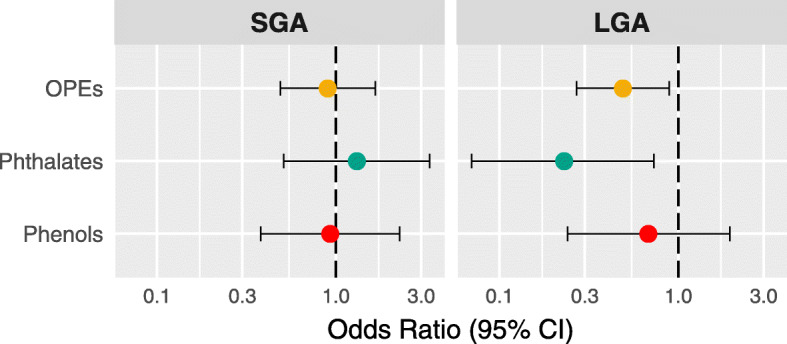
Adjusted OR (95% CI) of SGA and LGA associated with a one quartile increase in all exposure biomarkers within mixtures of OPE metabolites, phthalate metabolites, and phenols estimated using quantile-g computation models

Using quantile g-computation, we estimated the joint associations between mixtures of individual chemical classes (i.e., OPEs, phthalates, and phenols) and the odds of an SGA or LGA birth. The results from these multi-pollutant models were consistent with observations made from our single-pollutant models (Fig. 2; Additional File 1: Supplemental Table 9). Namely, we observed that simultaneously increasing both OPE metabolites by one quartile was associated with reduced odds of LGA (OR: 0.49 [95% CI: 0.27, 0.89]). Weights from the quantile g-computation model indicated that DPhP contributed more strongly than BDCPP to this association (Additional File 1: Supplemental Table 9). Increasing all phthalate metabolites by one quartile was also associated with reduced odds of LGA (OR: 0.23 [95% CI: 0.07, 0.73]). Within this model, MEP was assigned the largest negative weight (i.e., the greatest contribution to the negative partial effect). Finally, a one quartile increase in all phenols was associated with lower odds of LGA (OR: 0.68 [95% CI: 0.40, 1.94]), although the association did not reach statistical significance. As in the single-pollutant approach, the associations between exposure biomarker mixtures and SGA did not differ from the null.

## Discussion

In this case-control study, we investigated the association between prenatal exposure to consumer product chemicals and the odds of being born SGA or LGA, using both single- and multi-pollutant approaches. We observed inverse associations between individual biomarkers of exposure to OPEs, phthalates, and phenols and LGA births. Furthermore, results from our mixtures analyses showed potentially strong joint effects of overall chemical classes on odds of LGA births. Specifically, the overall mixture of OPE metabolites and that of phthalate metabolites was associated with 2–5 fold reductions in the odds of an LGA birth. Associations between consumer product chemicals and SGA were considerably more mixed and closer to the null. These results suggest that exposure to some consumer product chemicals may reduce fetal growth. However, because we did not observe a similar increase in the odds of SGA births, it is possible that effects may only be observed on the high end of the birth weight distribution.

This is among the first studies to examine the relationship between OPE exposure and fetal growth endpoints. We observed that levels of urinary DPhP as well as the mixture of OPE metabolites were associated with reduced odds of LGA births although there was no association with SGA. These findings are consistent with several other epidemiologic studies that indicate a potential link between OPE exposure and altered fetal growth [22, 23]. For example, a recent prospective case-control study of low birth weight (*n* = 339) based in China reported that the odds of low birth weight in women with the highest tertile of urinary DPhP was 4.62 (95% CI: 1.72, 12.40) times that observed in the lowest tertile [22]. Notably, concentrations of DPhP and BDCPP were higher in our cohort (DPhP median = 0.74 ng/mL, BDCPP median = 0.67 ng/mL) compared to the Chinese cohort (DPhP median = 0.05 ng/mL, BDCPP median = 0.05 ng/mL) [22]. In the US-based Pregnancy Infection and Nutrition Study (*n* = 349), OPE concentrations in maternal urine were higher (DPhP median = 1.31 ng/mL, BDCPP median = 1.85 ng/mL) compared to the present study and an inverse association between isopropyl-phenyl phenyl phosphate (ip-PPP) and birth weight was observed in females [23]. However, other recent US-based cohorts have reported null associations between OPE metabolites and fetal growth outcomes [55, 56].

Toxicologic evidence also supports the biologic plausibility of a relationship between OPE exposure and fetal growth. One study in mice demonstrated that in utero exposure to triphenyl phosphate (TPhP), the parent compound of DPhP, alters fetal and maternal liver insulin growth factor signaling, which is critical to the control of fetal growth and development [57, 58]. Additional research using a human placental cell line has also established that TPhP exposure increases both progesterone and human chorionic gonadotropin secretion via activation of the peroxisome proliferator-activated receptor gamma [59]. Changes to placental hormone production has important implications for both placental and fetal growth and development [58]. Taken together, these findings indicate the potential for prenatal OPE exposure to affect these processes.

Out of the 12 phthalate metabolites included in this analysis, MEP was most strongly associated with lower odds of LGA births both in single-pollutant models and within quantile g-computation models for the mixture of phthalate metabolites. In support of this observation, a previous prospective study based in China at the Ma’anshan Women and Children’s Health care Hospital (*n* = 3103) reported an inverse association between maternal urinary MEP concentrations, measured repeatedly across gestation, and birth weight [60]. However, the overall literature on prenatal phthalate exposure and fetal growth is inconsistent with respect to both the implicated phthalate species and the direction of effect [1]. This is also true for studies that have investigated the potential for sex-specific effects of phthalates in relation to fetal growth outcomes [1]. In this study, we observed suggestive sex-specific effects for several DEHP metabolites in relation to SGA births. Specifically, DEHP metabolites were associated with higher odds of SGA among female infants compared to males. However, this observation contrasts with the prevailing hypothesis that males are more susceptible to in utero environmental exposures, including DEHP, than females [61]. This hypothesis is driven by several characteristics of male pregnancies, such as slower maturation of male fetuses or the heightened maternal immune response to the Y-chromosome [61]. Additionally, the anti-androgenic effects of DEHP have been demonstrated to have sex-specific impacts on growth and development in males using animal models [62]. Previous work in the LIFECODES cohort (*n* = 482) did not observe associations between DEHP metabolites and birth weight z-scores, although reductions in ultrasound measures of fetal size were observed [63]. However, these associations did not differ according to fetal sex [63]. Given the small sample size and wide confidence intervals for our sex-specific results in this study, we caution against their over-interpretation.

Among the phenols included in this study, MPB and PPB were both highly detected in maternal urine and inversely associated with LGA. While quantile g-computation models did not identify a significant association with the mixture of phenols and LGA births (OR: 0.68 [95% CI: 0.40, 1.94]), MPB was assigned the largest weight for the partial negative effect in the mixture. Previous studies examining prenatal paraben exposure have reported inconsistent associations with fetal growth, with varying estimated directions and magnitude of effect [24–26, 64–66]. More broadly, however, parabens are suspected to have effects on body composition and altered weight [67–70]. For example, a study in the National Health and Nutrition Examination Survey (NHANES) reported that higher urinary parabens were associated with lower BMI in both children and adults [67]. Reports of pro- or anti-obesogenic properties of parabens may have relevance to our findings because LGA infants have disproportionately higher total body fat compared to AGA infants [71].

While we observed several inverse associations between consumer product chemicals and LGA births, our results with SGA were much more variable and closer to the null. Babies born LGA are larger than AGA babies in numerous respects, including birth weight, length, and head circumference. However, LGA infants are also characterized by a disproportionate increase in total body fat and a decrease in lean body mass [71]. This phenotypic difference suggests that whether a baby is born LGA is controlled, at least in part, by mechanisms related to maternal-fetal nutrient transfer and adipogenesis [72, 73]. Therefore, chemical exposures that impact these processes should be considered in relation to fetal overgrowth. On the other end of the spectrum, SGA captures a diverse set of infants that include both those who are pathologically small (i.e., growth restricted) and those who are constitutionally small [74, 75]. We expect that adverse chemical exposures during pregnancy would be related to growth restriction rather than small, but normal growth. However, infants experiencing pathological growth restriction may be a minority of infants classified as SGA, particularly when considering births at term (i.e., > 37 weeks gestation) [74]. Without being able to distinguish between these sets of infants, our SGA results may be biased towards the null. A better approach in future studies may be the use of a clinical definition for intrauterine growth restriction or fetal growth trajectories to appropriately isolate pathologically small infants from constitutionally small infants.

This study is not without other limitations. First, this study has a relatively small sample size. This prevented us from examining SGA births more closely to isolate growth restricted infants from constitutionally small infants. It also limited our ability to explore heterogeneity with greater precision or to examine non-linearity of the dose-response relationship. Second, we were lacking information on several potentially important confounders. For example, recent studies have shown that parabens sequester into adipocytes [76–78]. This sequestration may explain inverse associations between paraben exposure and body weight measures, including birth weight. In the context of this study, maternal adiposity is an important risk factor for LGA births [79, 80]. However, it is possible that we were unable to fully account for this potential confounding by solely adjusting for maternal pre-pregnancy BMI. A direct measure of body fat measured via methods such as bioelectrical impedance may better account for maternal adiposity. In addition, seasonality is another potential confounder as season has been shown to be a strong predictor of exposure to some consumer product chemicals, such as OPEs, and may be related to changes in fetal growth [54, 81]. Lastly, although this study is among the first to consider associations between OPEs and fetal growth, only two out of nine selected flame retardants (i.e. DPhP and BDCPP) were detected in maternal urine frequently enough for analysis. This is in part due to the analytical method used to quantify OPE metabolites, which was less sensitive for some metabolites relative to recent studies on this topic [22, 55, 56]. While this has limited our ability to examine associations between OPEs and fetal growth, it is worth pointing out that DPhP and BDCPP are two of the most frequently detected OPE metabolites in the general population [82].

Despite these limitations, this study also had several strengths relative to prior studies. First, we were able to assess prenatal exposure to consumer product chemicals using measurements from up to three study visits during pregnancy. As this study and others have demonstrated, the stability of consumer product chemical concentrations in urine is often low given their short half-lives [10, 11, 54]. Yet, many studies continue to rely on a single spot urine sample to assess prenatal exposure to these chemicals [1], and therefore, may be more susceptible to bias due to exposure misclassification [83]. Because this study uses averaged exposure measures from up to three study visits, it may be less susceptible to such misclassification. Second, this study examined consumer product chemical exposure in relation to both SGA and LGA births. While many studies have considered the potential for chemical exposures to reduce fetal growth, few include outcomes related to fetal overgrowth [1]. By including both SGA and LGA, this study identified associations at one end of the birth weight distribution (i.e., LGA), but not the other (i.e., SGA). Lastly, we went beyond single-pollutant models to estimate the joint effect of consumer product chemical mixtures on fetal growth [52]. Estimating the joint effects of consumer product chemicals is of particular interest because behavioral or regulatory changes to the use of consumer products would likely simultaneously affect exposure to classes of chemicals, rather than individual chemicals. Thus, joint effects correspond better to possible real-world actions, while simultaneously controlling for confounding between elements of the mixture.

## Conclusions

In this study, we observed that higher levels of exposure to several consumer product chemicals, including metabolites of OPEs and phthalates, were associated with lower odds of LGA births. Yet, findings were largely null for relationships between exposure biomarkers and SGA births. The lack of consistent association between these chemicals and SGA births may be the result of the small study sample or residual confounding. Nevertheless, these results may still be consistent with observations that many of these chemicals have been previously associated with reduced fetal growth metrics. Because findings are strongest when comparing LGA births to AGA births, they may suggest that associations between consumer product chemicals and birth weight are stronger at the high end of the birth weight distribution. However, future studies should more carefully examine the effects of consumer product chemical exposure across the full spectrum of birth weight to further investigate these associations.

## Supplementary Information

**Additional file 1.**

**Additional file 2.**

## Data Availability

The dataset analyzed during the current study are not publicly available due to the sensitive nature of human biological and environmental exposure data but are available from the corresponding author on reasonable request.

## Abbreviations

95% CI: 95% Confidence Interval
ACOG: American College of Obstetrics and Gynecology
AGA: Appropriate-for-gestational age
BDCPP: Bis (1,3-dichloro-2-propyl) phosphate
BMI: Body mass index
BWH: Brigham and Women’s Hospital
DEHP: Di-2-ethylhexyl phthalate
DPhP: Diphenyl phosphate
ICC: Intraclass correlation coefficient
IQR: Interquartile range
LGA: Large-for-gestational age
LOD: Limit of Detection
MECPP: Mono-(2-ethyl-5-carboxypentyl) phthalate
MEHHP: Mono-(2-ethyl-5-hydroxyhexyl) phthalate
MEHP: Mono-2-ethylhexyl phthalate
MEOHP: Mono-(2-ethyl-5-oxohexyl) phthalate
MEP: Mono-ethyl phthalate
MPB: Methyl paraben
NHANES: National Health and Examination Survey
OPE: Organophosphate ester
OR: Odds ratio
PPB: Propyl paraben
SGA: Small-for-gestational age
TPhP: Triphenyl phosphate
