# Supplementary material for: Prenatal exposure to consumer product chemical mixtures and size for gestational age at delivery
Source: Environ Health. 2021 Jun 10;20:68. doi: 10.1186/s12940-021-00724-z (PMC8194159; doi:10.1186/s12940-021-00724-z)
Supplement: Supplementary file 1 — Additional file 1. [file 12940_2021_724_MOESM1_ESM.docx]

**
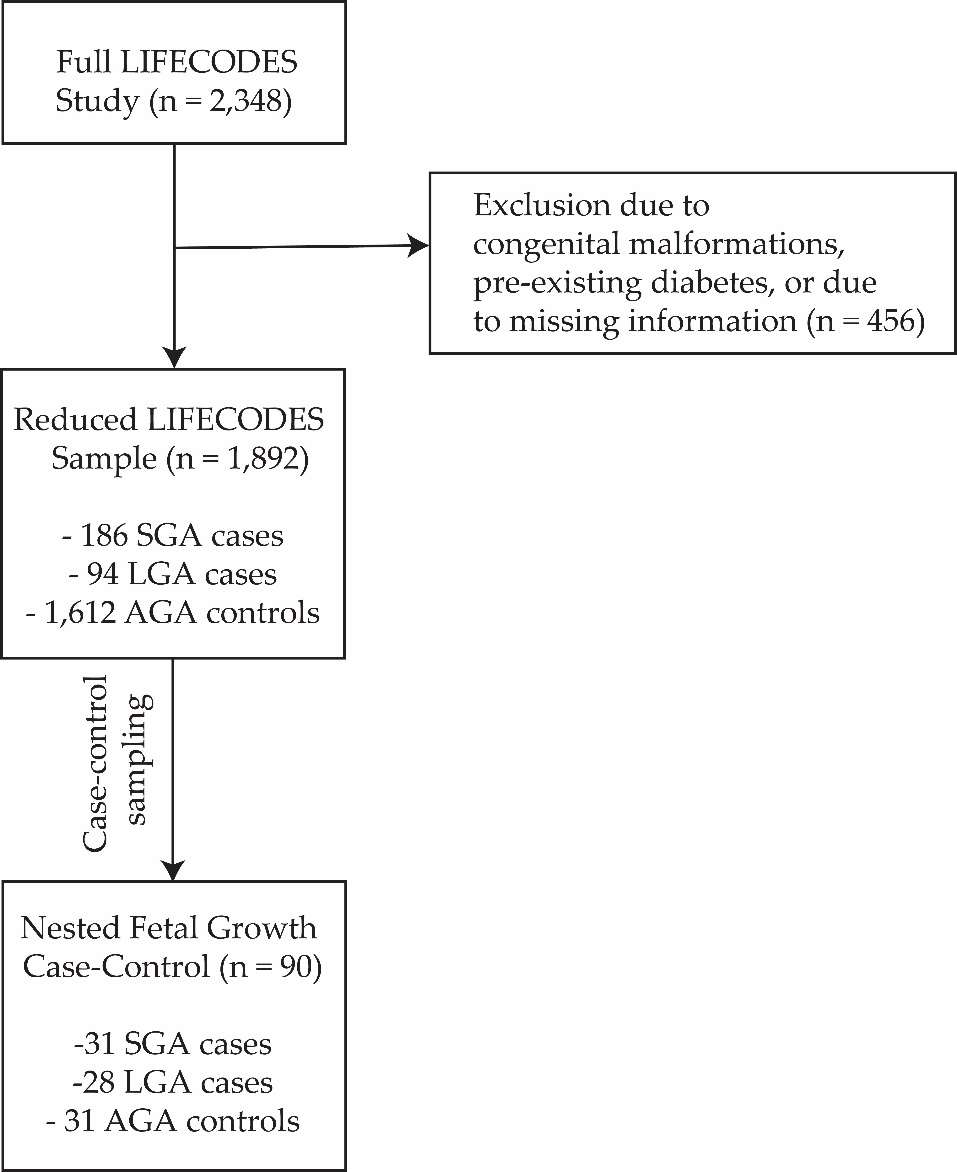
Supplemental Figure 1.** Flow-through diagram of study sampling and design for the nested case-control study.

| **Supplemental Table 1.** List of urinary analytes measured, their relation to parent compounds found in consumer products, and limits of detection for analysis (ng/mL). | | | | | | | |
| --- | --- | --- | --- | --- | --- | --- | --- |
| **Chemical**  **Class** | **Parent Compound(s)** | |  | **Measured Compound** | | | |
|  | **Abbreviation** | **Compound Name** |  | **Abbreviation** | **Compound Name** | **LOD**  **(ng/mL)** | **N^7^ (%)**  **< LOD** |
| OPEs^1^ | TCEtP | Tris(2-chloroethyl) phosphate |  | BCEtP | Bis(2-chloroethyl) phosphate | 2 | 248 (94) |
|  | TCPP | Tris(1-chloro-2-propyl) phosphate |  | BCPP | Bis(1-chloro-2-propyl) phosphate | 2 | 149 (57) |
|  | TDCPP | Tris(1,3-dichloro-2-propyl) phosphate |  | BDCPP | Bis(1,3-dichloro-2-propyl) phosphate | 0.1 | 25 (10) |
|  | TBuP | Tri-n-butyl phosphate |  | DBuP | Dibutyl phosphate | 0.5 | 262 (99) |
|  | TBzP | Tribenzyl phosphate |  | DBzP | Dibenzyl phosphate | 0.05 | 263 (100) |
|  | TPhP | Triphenyl phosphate |  | DPhP^3^ | Diphenyl phosphate^3^ | 0.1 | 8 (3) |
|  | ToCP | Tri-o-cresylphosphate |  | DoCP | Di-o-cresylphosphate | 0.1 | 262 (99) |
|  | TpCP | Tri-p-cresylphosphate |  | DpCp | Di-p-cresylphosphate | 0.1 | 241 (92) |
|  | EH-TBB | 2-Ethylhexyl-2,3,4,5-tebrabromobenzoate |  | TBBA | 2,3,4,5-Tetrabromobenzoic acid | 0.05 | 259 (98) |
| Phthalates | DEP | Diethyl phthalate |  | MEP | Mono-ethyl phthalate | 1 | 1 (0.4) |
|  | DBP | Dibutyl phthalate |  | MBP^4^ | Mono-n-butyl phthalate^4^ | 0.5 | 1 (0.4) |
|  | BzBP | Benzylbutyl phthalate |  | MBzP | Mono-benzyl phthalate | 0.2 | 4 (2) |
|  | DiBP | Di-isobutyl phthalate |  | MiBP | Mono-isobutyl phthalate | 0.1 | 1 (0.4) |
|  | DEHP | Di-2-ethylhexyl phthalate |  | MECPP | Mono-(2-ethyl-5-carboxypentyl) phthalate | 0.2 | 0 (0) |
|  |  |  |  | MEHHP | Mono-(2-ethyl-5-hydroxyhexyl) phthalate | 0.1 | 0 (0) |
|  |  |  |  | MEOHP | Mono-(2-ethyl-5-oxohexyl) phthalate | 0.1 | 0 (0) |
|  |  |  |  | MEHP | Mono-2-ethylhexyl phthalate | 1 | 94 (36) |
|  | DOP | Di-n-octyl phthalate |  | MCPP^5^ | Mono-(3-carboxypropyl) phthalate^5^ | 0.2 | 13 (5) |
|  | DiNP | Di-isonyl phthalate |  | MCOP | Mono-(carboxyoctyl) phthalate | 0.2 | 24 (9) |
|  | DiNP | Di-isonyl phthalate |  | MNP | Mono-isononyl phthalate | 0.5 | 228 (87) |
|  | DiDP | Di-isodecyl phthalate |  | MCNP | Mono-(carboxynonyl) phthalate | 0.2 | 103 (39) |
| Phenols^2^ | 2,4-D | 2,4-Dichlorophenoxyacetic acid |  | 2,4-DCP^6^ | 2,4-Dichlorophenol^6^ | 0.2 | 92 (35) |
|  | pDCB | para-Dichlorobenezene |  | 2,5-DCP^6^ | 2,5-Dichlorophenol^6^ | 0.2 | 46 (17) |
|  | BP3 | Benzophenone-3 |  | BP3 | Benzophenone-3 | 0.4 | 1 (0.4) |
|  | BPA | Bisphenol A |  | BPA | Bisphenol A | 0.4 | 83 (32) |
|  | BPF | Bisphenol F |  | BPF | Bisphenol F | 0.4 | 218 (83) |
|  | BPS | Bisphenol S |  | BPS | Bisphenol S | 0.4 | 199 (76) |
|  | BPB | Butyl paraben |  | BPB | Butyl paraben | 0.2 | 155 (59) |
|  | EPB | Ethyl paraben |  | EPB | Ethyl paraben | 1 | 132 (50) |
|  | MPB | Methyl paraben |  | MPB | Methyl paraben | 1 | 0 (0) |
|  | PPB | Propyl paraben |  | PPB | Propyl paraben | 0.2 | 5 (2) |
|  | TCS | Triclosan |  | TCS | Triclosan | 2 | 82 (31) |
|  | TCB | Triclocarban |  | TCB | Triclocarban | 2 | 253 (96) |
| ^1^EH-TBB is a not an OPE but is included in this category for ease of reporting.  ^2^Triclocarbon is not a phenol but is included this category for ease of reporting.  ^3^DPhP is a non-specific metabolite of other OPEs, including 2-ethylhexyl diphenyl phospate (EHDPHP), isopropylated triarylphosphate isomer (ITP), and tert-butylated triarylphosphate isomer (TBPP). ^.4^MBP is also a metabolite of benzylbutyl phthalate (BzBP). ^5^MCPP is also a metabolite of various other high molecular weight phthalates. ^6^2,4-DCP and 2,5-DCP are also metabolites of other chlorophenols and may be formed as disinfection byproducts during water treatment. ^7^n = 263 total samples across 3 study visits. | | | | | | | |

| **Supplemental Table 2.** Study population characteristics between participants from the overall LIFECODES study and the nested case-control study (N=1,892).^1^ | | | |
| --- | --- | --- | --- |
| **Characteristic** | **Overall LIFECODES participants (N=1,802)** | **Nested case-control study (n=90)** | **p^2^** |
| **Matched Variables** |  |  |  |
| Maternal age (years) | 32.5 (28.4, 36.4) | 33.2 (29.5, 37.3) | 0.23^a^ |
| Pre-pregnancy BMI (kg/m^2^) | 24.9 (21.6, 30.0) | 23.5 (21.4, 27.5) | 0.07^a^ |
| Race/Ethnicity |  |  |  |
| White | 971 (53.9%) | 54 (60.0%) | 0.06^b^ |
| Black | 269 (14.9%) | 18 (20.0%) |  |
| Other^3^ | 562 (31.2%) | 18 (20.0%) |  |
| Gestational Age (weeks) | 39.0 (37.6, 39.7) | 38.7 (37.9, 39.4) | 0.65^a^ |
| Size for gestational age at delivery |  |  |  |
| Small | 186 (10.3%) | 31 (34.4%) | <0.01^b^ |
| Appropriate | 1522 (84.5%) | 31 (34.4%) |  |
| Large | 94 (5.2%) | 28 (31.1%) |  |
| ^1^ Data given as n (%) or median (25^th^, 75^th^ percentiles)  ^2^ *p*-Values represent comparisons between overall and nested case-control groups from: a) Wilcoxon rank-sum tests; b) Pearson’s chi-squared test; or c) Fisher’s exact test.  ^3^ The “Other” race/ethnicity category is a condensation of larger categories with insufficient sample size for disaggregation (e.g., Hispanic/Latino ethnicity, South Asian, East Asian, Native American, multiracial). | | | |

| **Supplemental Table 3.** Median (25^th^, 75^th^ percentile) and detection frequencies for uncorrected average exposure biomarker concentrations (ng/mL) in the overall study population and according to case status. | | | | | | | | | |  |
| --- | --- | --- | --- | --- | --- | --- | --- | --- | --- | --- |
| **Chemical  Class** | **Urinary Analyte (ng/mL)** | **Overall (n = 90)** | | **SGA (n = 31)** | | **AGA (n = 31)** | | **LGA (n = 28)** | | |
|  |  | **N (%)**  **< LOD^1^** | **Median**  **(25^th^, 75^th^)** | **N (%)**  **< LOD^1^** | **Median**  **(25^th^, 75th)** | **N (%)**  **< LOD^1^** | **Median**  **(25^th^, 75th)** | **N (%)**  **< LOD^1^** | **Median**  **(25^th^, 75th)** | |
| OPEs | BDCPP | 25 (10) | 0.63 (0.33, 1.25) | 9 (11) | 0.69 (0.34, 1.43) | 7 (8) | 0.54 (0.32, 1.52) | 9 (10) | 0.60 (0.30, 0.86) | |
|  | DPhP | 8 (3) | 0.62 (0.40, 1.48) | 3 (4) | 0.65 (0.34, 2.06) | 3 (3) | 0.75 (0.46, 1.48) | 2 (2) | 0.54 (0.40, 0.92) | |
| Phthalates | MEP | 1 (0.4) | 46.7 (11.8, 109) | 1 (1) | 32.5 (7.71, 203) | 0 (0) | 73.4 (29.5, 119) | 0 (0) | 21.8 (8.64, 58.6) | |
|  | MBP | 1 (0.4) | 8.93 (4.84, 16.4) | 0 (0) | 9.12 (4.81, 19.9) | 0 (0) | 9.95 (4.17, 16.5) | 1 (1) | 6.91 (4.99, 12.1) | |
|  | MBzP | 4 (2) | 2.74 (1.16, 7.44) | 2 (2) | 2.80 (1.15, 7.83) | 0 (0) | 2.15 (1.16, 9.44) | 2 (2) | 3.18 (1.24, 5.46) | |
|  | MiBP | 1 (0.4) | 5.35 (2.78, 9.65) | 0 (0) | 6.06 (3.20, 11.2) | 0 (0) | 6.29 (2.26, 12.4) | 1 (1) | 4.89 (2.46, 7.72) | |
|  | MECPP | 0 (0) | 8.15 (4.88, 17.8) | 0 (0) | 10.0 (5.33, 16.5) | 0 (0) | 7.63 (4.88, 18.7) | 0 (0) | 6.54 (3.69, 16.1) | |
|  | MEHHP | 0 (0) | 6.07 (3.50, 11.0) | 0 (0) | 7.28 (3.99, 11.5) | 0 (0) | 4.95 (3.58, 10.8) | 0 (0) | 5.03 (3.13, 10.5) | |
|  | MEOHP | 0 (0) | 4.10 (2.41, 7.80) | 0 (0) | 5.44 (2.48, 7.05) | 0 (0) | 3.69 (2.41, 8.27) | 0 (0) | 3.42 (2.16, 7.48) | |
|  | MEHP | 94 (36) | 1.45 (0.90, 3.24) | 29 (34) | 1.62 (1.15, 3.43) | 32 (36) | 1.32 (0.86, 3.50) | 33 (37) | 1.42 (0.84, 2.53) | |
|  | MCPP | 13 (5) | 2.04 (0.91, 4.87) | 4 (5) | 1.94 (0.77, 6.51) | 8 (9) | 2.30 (1.24, 6.10) | 1 (1) | 2.07 (0.81, 4.32) | |
|  | MCOP | 24 (9) | 1.96 (0.77, 5.28) | 6 (7) | 2.22 (0.76, 7.20) | 11 (13) | 2.02 (1.03, 5.53) | 7 (8) | 1.76 (0.57, 5.00) | |
|  | MCNP | 103 (39) | 0.26 (0.18, 0.44) | 35 (41) | 0.26 (0.15, 0.45) | 36 (41) | 0.31 (0.21, 0.47) | 32 (36) | 0.21 (0.13, 0.40) | |
| Phenols | 2,4-DCP | 92 (35) | 0.29 (0.17, 0.49) | 31 (36) | 0.25 (0.18, 0.50) | 28 (32) | 0.29 (0.15, 0.45) | 33 (37) | 0.29 (0.21, 0.56) | |
|  | 2.5-DCP | 46 (17) | 0.60 (0.30, 1.45) | 16 (19) | 0.71 (0.42, 1.50) | 14 (16) | 0.60 (0.31, 1.45) | 16 (18) | 0.38 (0.24, 0.96) | |
|  | BP3 | 1 (0.4) | 28.2 (12.0, 72.2) | 1 (1) | 35.0 (9.78, 70.5) | 0 (0) | 23.9 (12.6, 72.2) | 0 (0) | 33.8 (12.2, 130) | |
|  | BPA | 83 (32) | 0.61 (0.34, 0.90) | 0 (0) | 0.63 (0.32, 0.90) | 1 (1) | 0.52 (0.33, 0.88) | 0 (0) | 0.64 (0.41, 0.99) | |
|  | MPB | 0 (0) | 80.8 (28.7, 183) | 0 (0) | 106 (38.4, 255) | 0 (0) | 111 (57.0, 178) | 0 (0) | 40.4 (20.6, 154) | |
|  | PPB | 5 (2) | 14.7 (5.84, 46.2) | 1 (1) | 12.1 (5.84, 68.4) | 3 (3) | 23.2 (10.2, 51.4) | 1 (1) | 8.31 (4.10, 27.4) | |
|  | TCS | 82 (31) | 5.80 (2.22, 44.8) | 30 (35) | 3.57 (1.16, 55.2) | 22 (25) | 4.51 (1.59, 13.7) | 30 (33) | 10.8 (3.54, 48.6) | |
| n = 263 total samples: 85 SGA samples, 88 LGA samples, and 90 AGA samples across 3 study visits. | | | | | | | | | | |

| **Supplemental Table 4**. Median participant average exposure biomarker concentrations (ng/mL) across demographic characteristics. | | | | | | | | | | | | | | | | | | | | |
| --- | --- | --- | --- | --- | --- | --- | --- | --- | --- | --- | --- | --- | --- | --- | --- | --- | --- | --- | --- | --- |
|  | **BDCPP** | **DPhP** | **MEP** | **MBP** | **MBzP** | **MiBP** | **MECPP** | **MEHHP** | **MEOHP** | **MEHP** | **MCPP** | **MCOP** | **MCNP** | **2,4-DCP** | **2,5-DCP** | **BP3** | **BPA** | **MPB** | **PPB** | **TCS** |
| **Age, years** |  |  |  |  | ** | ** |  |  |  |  |  |  | ** |  |  | ** |  |  |  | * |
| < 25 | 0.74 | 0.82 | 93.2 | 8.15 | 4.29 | 4.77 | 13.2 | 7.98 | 4.60 | 1.83 | 2.18 | 4.06 | 0.30 | 0.24 | 0.52 | 9.50 | 0.68 | 164 | 21.5 | 1.77 |
| 25 - 30 | 0.77 | 0.72 | 111 | 11.8 | 6.24 | 8.12 | 11.7 | 8.40 | 6.17 | 1.86 | 1.85 | 1.51 | 0.23 | 0.46 | 0.80 | 20.8 | 0.60 | 124 | 27.0 | 5.46 |
| 30 - 35 | 0.66 | 0.74 | 35.2 | 9.20 | 2.68 | 4.68 | 8.32 | 5.08 | 3.52 | 1.50 | 2.00 | 1.89 | 0.27 | 0.31 | 0.57 | 29.2 | 0.75 | 82.6 | 15.5 | 14.4 |
| 35+ | 0.67 | 0.76 | 41.1 | 9.86 | 1.99 | 5.75 | 9.28 | 6.69 | 4.21 | 2.45 | 2.79 | 3.01 | 0.37 | 0.31 | 0.65 | 55.8 | 0.51 | 101 | 18.6 | 6.32 |
|  |  |  |  |  |  |  |  |  |  |  |  |  |  |  |  |  |  |  |  |  |
| **Pre-pregnancy BMI, kg/m^2^** | * |  |  | ** | ** | ** |  |  |  |  | * |  |  |  |  |  |  |  |  |  |
| < 25 | 0.47 | 0.72 | 38.1 | 8.17 | 2.47 | 5.04 | 8.59 | 5.64 | 4.07 | 1.77 | 2.13 | 1.72 | 0.31 | 0.28 | 0.65 | 39.1 | 0.58 | 105 | 21.3 | 5.52 |
| 25 - 30 | 0.84 | 0.72 | 52.0 | 12.7 | 3.71 | 7.32 | 9.90 | 7.38 | 5.32 | 2.31 | 1.88 | 2.01 | 0.28 | 0.36 | 0.59 | 30.4 | 0.67 | 125 | 17.5 | 10.7 |
| > 30 | 0.80 | 0.98 | 48.0 | 11.0 | 5.69 | 7.80 | 11.5 | 6.47 | 4.91 | 1.54 | 3.95 | 3.76 | 0.33 | 0.42 | 0.86 | 36.5 | 0.73 | 91.1 | 13.4 | 6.60 |
|  |  |  |  |  |  |  |  |  |  |  |  |  |  |  |  |  |  |  |  |  |
| **Maternal Insurance** | ** |  | ** | ** | ** | ** | ** | * | ** |  |  |  |  |  |  | * |  |  |  |  |
| Private | 0.56 | 0.72 | 37.0 | 8.55 | 2.51 | 5.11 | 8.55 | 5.78 | 3.95 | 1.76 | 2.07 | 2.01 | 0.30 | 0.30 | 0.58 | 44.8 | 0.62 | 100 | 18.8 | 8.70 |
| Public | 0.95 | 0.82 | 57.0 | 13.0 | 6.85 | 8.17 | 14.2 | 9.13 | 5.77 | 2.36 | 3.19 | 2.07 | 0.31 | 0.37 | 0.96 | 16.2 | 0.67 | 131 | 22.5 | 2.61 |
|  |  |  |  |  |  |  |  |  |  |  |  |  |  |  |  |  |  |  |  |  |
| **Maternal Race** |  |  | ** |  | ** | ** |  |  |  |  |  | * | ** | ** | ** | * | * | ** | * | * |
| White | 0.53 | 0.70 | 35.5 | 8.47 | 2.59 | 5.05 | 8.71 | 5.78 | 3.95 | 1.75 | 2.24 | 2.26 | 0.31 | 0.28 | 0.58 | 50.7 | 0.58 | 79.5 | 14.6 | 6.62 |
| Black | 0.95 | 0.87 | 119 | 14.6 | 7.63 | 6.79 | 15.05 | 9.18 | 7.02 | 2.39 | 2.74 | 1.96 | 0.31 | 0.59 | 2.14 | 24.2 | 0.82 | 184 | 24.9 | 21.7 |
| Other | 0.74 | 0.85 | 35.6 | 10.2 | 4.32 | 8.16 | 8.88 | 6.76 | 4.56 | 1.98 | 1.67 | 1.65 | 0.24 | 0.24 | 0.51 | 16.2 | 0.60 | 124 | 32.3 | 1.99 |
|  |  |  |  |  |  |  |  |  |  |  |  |  |  |  |  |  |  |  |  |  |
| **Maternal Education** |  |  |  | ** | ** | ** | * |  |  |  |  |  |  |  |  | ** |  |  |  | ** |
| ≤ High School | 1.72 | 0.76 | 188 | 17.7 | 5.01 | 9.79 | 15.3 | 9.33 | 5.76 | 2.46 | 3.93 | 4.48 | 0.31 | 0.22 | 0.60 | 12.3 | 0.69 | 172 | 19.2 | 1.55 |
| Some college/technical school | 0.82 | 0.76 | 49.4 | 12.9 | 7.89 | 8.12 | 10.3 | 7.47 | 5.55 | 1.76 | 2.28 | 1.88 | 0.28 | 0.37 | 0.74 | 11.2 | 0.70 | 108 | 16.8 | 7.92 |
| ≥ College graduate | 0.54 | 0.73 | 38.2 | 8.52 | 2.26 | 5.16 | 8.59 | 5.54 | 3.82 | 1.77 | 2.00 | 2.01 | 0.30 | 0.32 | 0.59 | 54.0 | 0.60 | 101 | 19.5 | 8.18 |
|  |  |  |  |  |  |  |  |  |  |  |  |  |  |  |  |  |  |  |  |  |
| **Parity** |  |  | * |  |  |  |  |  |  |  |  |  |  |  |  | * | ** |  |  |  |
| Nulliparous | 0.56 | 0.72 | 92.0 | 9.24 | 3.63 | 5.67 | 11.7 | 7.20 | 4.68 | 2.16 | 2.28 | 2.49 | 0.30 | 0.42 | 0.56 | 26.0 | 0.77 | 134 | 21.5 | 6.41 |
| Parous | 0.78 | 0.77 | 41.0 | 9.62 | 2.66 | 5.69 | 8.55 | 6.23 | 4.44 | 1.79 | 2.22 | 1.95 | 0.30 | 0.29 | 0.72 | 44.8 | 0.58 | 104 | 18.8 | 6.30 |
|  |  |  |  |  |  |  |  |  |  |  |  |  |  |  |  |  |  |  |  |  |
| **Smoking During Pregnancy** |  |  | ** |  | * |  | * | * | * |  |  |  |  |  | * | ** |  | * |  |  |
| No | 0.67 | 0.75 | 41.0 | 9.68 | 2.96 | 5.69 | 8.71 | 6.15 | 4.26 | 1.82 | 2.28 | 2.01 | 0.30 | 0.31 | 0.59 | 43.2 | 0.61 | 103 | 18.2 | 6.30 |
| Yes | 0.72 | 0.52 | 151 | 7.74 | 5.77 | 5.47 | 18.7 | 10.9 | 7.41 | 2.29 | 2.09 | 2.80 | 0.29 | 0.61 | 2.09 | 3.46 | 0.83 | 307 | 64.9 | 28.0 |
|  |  |  |  |  |  |  |  |  |  |  |  |  |  |  |  |  |  |  |  |  |
| **Alcohol Use During Pregnancy** |  |  |  |  |  | * |  |  |  |  | * | * | * | * |  | * |  |  |  | * |
| No | 0.66 | 0.74 | 41.9 | 9.51 | 3.57 | 5.69 | 8.94 | 6.31 | 4.44 | 1.89 | 2.16 | 1.95 | 0.30 | 0.30 | 0.60 | 29.4 | 0.61 | 109 | 19.3 | 5.85 |
| Yes | 0.95 | 1.16 | 86.5 | 9.50 | 2.04 | 3.21 | 12.0 | 9.48 | 5.92 | 3.01 | 5.31 | 4.74 | 0.57 | 0.56 | 0.68 | 69.3 | 0.83 | 86.2 | 15.4 | 101 |
|  |  |  |  |  |  |  |  |  |  |  |  |  |  |  |  |  |  |  |  |  |
| **Fetal Sex** |  |  |  |  |  |  |  | * | * |  |  |  |  |  |  |  |  |  |  |  |
| Female | 0.78 | 0.77 | 49.4 | 9.23 | 2.74 | 5.47 | 8.50 | 5.30 | 3.92 | 1.73 | 1.95 | 1.71 | 0.29 | 0.28 | 0.70 | 42.8 | 0.67 | 115 | 18.2 | 7.43 |
| Male | 0.62 | 0.73 | 41.0 | 10.2 | 3.50 | 6.19 | 10.9 | 7.28 | 5.75 | 2.29 | 2.63 | 2.07 | 0.31 | 0.33 | 0.59 | 27.9 | 0.60 | 93.9 | 19.4 | 4.88 |
| Asterisks indicate Kruskal-Wallis Test p-value: ** < 0.05; * < 0.1. | | | | | | | | | | | | | | | | | | | | |

| **Supplemental Table 5.** Intraclass correlation coefficients (ICC [95% CI]) for exposure biomarker concentrations across study visits. | | |
| --- | --- | --- |
| **Chemical Class** | **Urinary Analyte** | **ICC (95% CI)** |
| OPEs | BDCPP | 0.38 (0.26, 0.52) |
|  | DPhP | 0.29 (0.18, 0.44) |
| Phthalates | MEP | 0.63 (0.52, 0.72) |
|  | MBP | 0.60 (0.49, 0.70) |
|  | MBzP | 0.74 (0.65, 0.81) |
|  | MiBP | 0.59 (0.48, 0.70) |
|  | MECCP | 0.34 (0.22, 0.48) |
|  | MEHHP | 0.31 (0.19, 0.45) |
|  | MEOHP | 0.35 (0.23, 0.49) |
|  | MEHP | 0.34 (0.22, 0.48) |
|  | MCPP | 0.30 (0.18, 0.45) |
|  | MCOP | 0.25 (0.13, 0.41) |
|  | MCNP | 0.30 (0.19, 0.45) |
| Phenols | 2,4-DCP | 0.45 (0.33, 0.58) |
|  | 2,5-DCP | 0.47 (0.35, 0.60) |
|  | BP3 | 0.54 (0.43, 0.66) |
|  | BPA | 0.33 (0.21, 0.48) |
|  | MPB | 0.55 (0.43, 0.66) |
|  | PPB | 0.31 (0.19, 0.45) |
|  | TCS | 0.59 (0.48, 0.70) |

| **Supplemental Table 6.** Unadjusted OR (95% CI) of SGA and LGA associated with an IQR-increase in average urinary exposure biomarker concentrations. | | | | |
| --- | --- | --- | --- | --- |
| **Chemical Class** | **Urinary Analyte** | **SGA** |  | **LGA** |
|  |  | **OR (95% CI)** |  | **OR (95% CI)** |
| OPEs | BDCPP | 1.06 (0.57, 2.00) |  | 0.61 (0.32, 1.16) |
|  | DPhP | 0.99 (0.56, 1.76) |  | 0.48 (0.24, 0.96)* |
| Phthalates | MEP | 0.61 (0.31, 1.20) |  | 0.42 (0.19, 0.90)* |
|  | MBP | 1.16 (0.76, 1.75) |  | 0.73 (0.45, 1.20) |
|  | MBzP | 1.22 (0.69, 2.17) |  | 0.90 (0.49, 1.66) |
|  | MiBP | 1.04 (0.61, 1.77) |  | 0.63 (0.34, 1.19) |
|  | MECPP | 1.05 (0.51, 2.19) |  | 0.70 (0.32, 1.51) |
|  | MEHHP | 1.32 (0.69, 2.54) |  | 0.87 (0.43, 1.75) |
|  | MEOHP | 1.21 (0.60, 2.45) |  | 0.87 (0.41, 1.82) |
|  | MEHP | 1.14 (0.61, 2.15) |  | 0.74 (0.37, 1.47) |
|  | MCPP | 0.83 (0.45, 1.55) |  | 0.64 (0.33, 1.24) |
|  | MCOP | 0.86 (0.38, 1.93) |  | 0.60 (0.26, 1.38) |
|  | MCNP | 0.88 (0.50, 1.55) |  | 0.62 (0.33, 1.17) |
| Phenols | 2,4-DCP | 1.15 (0.60, 2.20) |  | 1.27 (0.66, 2.44) |
|  | 2,5-DCP | 1.00 (0.59, 1.69) |  | 0.81 (0.45, 1.45) |
|  | BP3 | 0.79 (0.42, 1.47) |  | 0.99 (0.53, 1.85) |
|  | BPA | 1.01 (0.58, 1.75) |  | 1.35 (0.77, 2.37) |
|  | MPB | 0.89 (0.45, 1.73) |  | 0.36 (0.17, 0.79)* |
|  | PPB | 0.80 (0.39, 1.61) |  | 0.44 (0.21, 0.92)* |
|  | TCS | 1.13 (0.49, 2.56) |  | 1.99 (0.87, 4.58) |
| Asterisks indicate p < 0.05. | | | | |

| **Supplemental Table 7.** Adjusted**^1^** OR (95% CI) of SGA and LGA associated with an IQR-increase in urinary average exposure biomarkers within strata of fetal sex. | | | | | | | | |
| --- | --- | --- | --- | --- | --- | --- | --- | --- |
| **Chemical Class** | **Urinary**  **Analyte** | **SGA** | | |  | **LGA** | | |
|  |  | **Female** | **Male** | **p^2^** |  | **Female** | **Male** | **p^2^** |
| OPEs | BDCPP | 1.05 (0.50, 2.23) | 1.36 (0.37, 4.97) | 0.73 |  | 0.78 (0.34, 1.79) | 0.24 (0.06, 1.01) | 0.15 |
|  | DPhP | 1.26 (0.58, 2.74) | 0.72 (0.27, 1.97) | 0.38 |  | 0.64 (0.25, 1.61) | 0.16 (0.03, 0.74)* | 0.12 |
| Phthalates | MEP | 0.68 (0.25, 1.79) | 0.44 (0.14, 1.34) | 0.57 |  | 0.29 (0.08, 0.98)* | 0.36 (0.12, 1.12) | 0.77 |
|  | MBP | 1.37 (0.77, 2.45) | 0.99 (0.42, 2.33) | 0.53 |  | 0.58 (0.25, 1.35) | 0.81 (0.36, 1.82) | 0.56 |
|  | MBzP | 1.25 (0.54, 2.89) | 1.66 (0.54, 5.05) | 0.67 |  | 1.04 (0.41, 2.68) | 0.94 (0.30, 2.96) | 0.88 |
|  | MiBP | 1.33 (0.59, 2.99) | 0.91 (0.36, 2.34) | 0.53 |  | 0.40 (0.13, 1.24) | 0.65 (0.25, 1.70) | 0.50 |
|  | MECPP | 2.13 (0.70, 6.54) | 0.50 (0.15, 1.65) | 0.08 |  | 0.80 (0.22, 2.87) | 0.48 (0.16, 1.48) | 0.56 |
|  | MEHHP | 3.10 (1.04, 9.24)* | 0.63 (0.22, 1.80) | 0.04 |  | 1.44 (0.44, 4.79) | 0.51 (0.18, 1.44) | 0.20 |
|  | MEOHP | 2.26 (0.75, 6.79) | 0.67 (0.21, 2.13) | 0.13 |  | 1.29 (0.37, 4.54) | 0.53 (0.17, 1.58) | 0.28 |
|  | MEHP | 3.84 (1.26, 11.7)* | 0.35 (0.11, 1.09) | <0.01 |  | 1.57 (0.49, 5.10) | 0.37 (0.12, 1.09) | 0.07 |
|  | MCPP | 1.01 (0.45, 2.24) | 0.53 (0.15, 1.84) | 0.39 |  | 0.57 (0.22, 1.46) | 0.51 (0.15, 1.68) | 0.88 |
|  | MCOP | 1.15 (0.39, 3.38) | 0.54 (0.14, 2.15) | 0.40 |  | 0.40 (0.12, 1.37) | 0.76 (0.20, 2.84) | 0.49 |
|  | MCNP | 1.69 (0.68, 4.17) | 0.38 (0.12, 1.23) | 0.05 |  | 0.99 (0.37, 2.71) | 0.23 (0.07, 0.80) | 0.07 |
| Phenols | 2,4-DCP | 1.14 (0.38, 3.41) | 1.18 (0.46, 3.05) | 0.96 |  | 1.04 (0.30, 3.58) | 1.40 (0.56, 3.51) | 0.68 |
|  | 2,5-DCP | 1.00 (0.40, 2.48) | 0.95 (0.42, 2.14) | 0.93 |  | 0.45 (0.13, 1.52) | 0.83 (0.37, 1.84) | 0.37 |
|  | BP3 | 0.96 (0.39, 2.40) | 0.49 (0.15, 1.59) | 0.36 |  | 0.76 (0.28, 2.10) | 0.79 (0.29, 2.15) | 0.95 |
|  | BPA | 1.17 (0.59, 2.33) | 0.59 (0.19, 1.85) | 0.31 |  | 1.83 (0.86, 3.93) | 0.78 (0.26, 2.37) | 0.21 |
|  | MPB | 1.08 (0.33, 3.53) | 0.81 (0.32, 2.06) | 0.70 |  | 0.09 (0.02, 0.45)* | 0.41 (0.15, 1.17) | 0.11 |
|  | PPB | 1.02 (0.36, 2.85) | 0.62 (0.21, 1.82) | 0.51 |  | 0.19 (0.05, 0.74)* | 0.44 (0.15, 1.31) | 0.32 |
|  | TCS | 1.35 (0.41, 4.42) | 0.82 (0.21, 3.25) | 0.59 |  | 2.29 (0.64, 8.23) | 1.64 (0.48, 5.65) | 0.71 |
| Asterisks indicate p < 0.05.  ^1^Adjusted for age (years), pre-pregnancy BMI (kg/m^2^), maternal race (white/Black/other), maternal education (high school or less/some college or technical school/completed college or greater).  ^2^Wald p-value for interaction. | | | | | | | | |

| **Supplemental Table 8.** Adjusted^1^ OR (95% CI) for SGA and LGA associated with IQR-increase in average exposure biomarker concentrations excluding women reporting the use of cigarettes and alcohol. | | | | |
| --- | --- | --- | --- | --- |
| **Chemical Class** | **Urinary Analyte** | **SGA** |  | **LGA** |
|  |  | **aOR (95% CI)** |  | **aOR (95% CI)** |
| OPEs | BDCPP | 0.90 (0.42, 1.96) |  | 0.42 (0.18, 0.97)* |
|  | DPhP | 1.01 (0.52, 1.96) |  | 0.35 (0.15, 0.80)* |
| Phthalates | MEP | 0.39 (0.16, 0.96)* |  | 0.36 (0.14, 0.89)* |
|  | MBP | 1.14 (0.61, 2.12) |  | 0.72 (0.39, 1.33) |
|  | MBzP | 1.21 (0.56, 2.58) |  | 0.98 (0.44, 2.17) |
|  | MiBP | 1.15 (0.55, 2.40) |  | 0.56 (0.25, 1.24) |
|  | MECPP | 0.88 (0.37, 2.10) |  | 0.67 (0.28, 1.61) |
|  | MEHHP | 1.28 (0.58, 2.79) |  | 0.99 (0.45, 2.17) |
|  | MEOHP | 1.27 (0.55, 2.94) |  | 0.99 (0.43, 2.29) |
|  | MEHP | 1.24 (0.59, 2.59) |  | 0.81 (0.38, 1.74) |
|  | MCPP | 0.77 (0.37, 1.63) |  | 0.58 (0.26, 1.26) |
|  | MCOP | 0.75 (0.30, 1.90) |  | 0.58 (0.23, 1.49) |
|  | MCNP | 0.88 (0.42, 1.81) |  | 0.54 (0.25, 1.19) |
| Phenols | 2,4-DCP | 1.76 (0.68, 4.51) |  | 1.71 (0.68, 4.27) |
|  | 2,5-DCP | 1.12 (0.53, 2.35) |  | 0.75 (0.35, 1.61) |
|  | BP3 | 1.23 (0.53, 2.83) |  | 0.98 (0.43, 2.25) |
|  | BPA | 0.98 (0.54, 1.80) |  | 1.39 (0.76, 2.56) |
|  | MPB | 0.98 (0.40, 2.44) |  | 0.30 (0.11, 0.79)* |
|  | PPB | 0.70 (0.29, 1.65) |  | 0.36 (0.15, 0.89)* |
|  | TCS | 2.03 (0.67, 6.14) |  | 3.53 (1.17, 10.6)* |
| Asterisks indicate p < 0.05.  Abbreviations: IQR = interquartile range; aOR = adjusted odds ratio  ^1^Adjusted for age (years), pre-pregnancy BMI (kg/m^2^), maternal race (white/Black/other), maternal education (high school or less/some college or technical school/completed college or greater), and fetal sex (female/male). | | | | |
|  |  |  |  |  |

| **Supplemental Table 9.** Adjusted^1^ OR (95% CI) for the joint effect of (1) OPE, (2) phthalate, and (3) phenol mixtures on LGA births and the weights representing the proportion of positive or negative partial effects for each exposure biomarker in quantile g-computation models. | | | | |
| --- | --- | --- | --- | --- |
| **Mixture Model** | **Urinary Analyte** | **Weights** | | **aOR (95% CI)** |
|  |  | **Negative Weights** | **Positive Weights** |  |
| OPEs | DPhP | 0.74 |  | 0.49 (0.27, 0.89)* |
|  | BDCPP | 0.26 |  |  |
| Phthalates | MEP | 0.43 |  | 0.23 (0.07, 0.73)* |
|  | MCNP | 0.19 |  |  |
|  | MCPP | 0.15 |  |  |
|  | MBP | 0.13 |  |  |
|  | MCOP | 0.04 |  |  |
|  | MiBP | 0.03 |  |  |
|  | MEHP | 0.01 |  |  |
|  | MEOHP |  | 0.08 |  |
|  | MECPP |  | 0.15 |  |
|  | MEHHP |  | 0.26 |  |
|  | MBzP |  | 0.50 |  |
| Phenols | MPB | 0.43 |  | 0.68 (0.40, 1.94) |
|  | 2,5-DCP | 0.30 |  |  |
|  | PPB | 0.27 |  |  |
|  | 2,4-DCP |  | 0.07 |  |
|  | BPA |  | 0.16 |  |
|  | BP3 |  | 0.30 |  |
|  | TCS |  | 0.46 |  |
| Asterisks indicate p < 0.05.  Abbreviations: aOR = adjusted odds ratio  ^1^Adjusted for age (years), pre-pregnancy BMI (kg/m^2^), maternal race (white/Black/other), maternal education (high school or less/some college or technical school/completed college or greater), and fetal sex (female/male). | | | | |
