# Supplementary material for: Prenatal exposure to consumer product chemical mixtures and size for gestational age at delivery
Source: Environ Health. 2021 Jun 10;20:68. doi: 10.1186/s12940-021-00724-z (PMC8194159; doi:10.1186/s12940-021-00724-z)
Supplement: Supplementary file 2 — Additional file 2. [file 12940_2021_724_MOESM2_ESM.docx]

**Appendix 1. Methods for analysis of organophosphate ester flame retardant metabolites.**

Nine flame retardant metabolites were measured in previously unthawed urine samples by NSF International (Ann Arbor, MI) using isotope dilution–liquid chromatography–tandem mass spectrometry (ID–LC–MS/MS): three chlorinated diakyl phosphates, diphenyl phosphate, 2,3,4,5-tetrabromobenzoic acid, and four other organophosphates. The method had been developed to simulate The Centers for Disease Control and Prevention (CDC) analytical method [1]. The methods were validated in accordance with Food and Drug Administration Guidance for Industry: Bioanalytical Method Validation [2].

Samples underwent enzymatic deconjugation of glucuronidated species, off-line solid phase extraction, and analysis using a Thermo Scientific (Waltham, MA, USA) Quantiva triple quadrupole mass spectrometer using multiple reaction monitoring with heated electrospray ionization in negative mode. Urine sample off-line extraction was performed using Phenomenex (Torrance, CA, USA) Strata X-AW 33μm Polymeric Weak Anion 60mg/well, 96 well plate. Chromatographic separation was performed using an Agilent (Santa Clara, CA, USA) ZORBAX Eclipse XDB-C8 5μm (3.0 x 150mm) analytical column. Flame retardant calibration ranges utilized were 0.2 – 160 ng/ml. The validated analyte calibration curve correlation coefficient (R^2^) range was 0.985-0.999. The method accuracy (% nominal concentration) and precision (%RSD) were determined through six replicate analyses of analytes spiked at two or three different concentrations in human urine across validation runs on three separate days (n =18) which reflects both the intra-day and inter-day variability of the assay. The accuracy (% nominal concentration) range across all analytes was 67–124% with precision (%RSD) range for urine quality control samples across all analytes being 1.8–14%.

1. Jayatilaka, N.K., et al., *Quantification of three chlorinated dialkyl phosphates, diphenyl phosphate, 2,3,4,5-tetrabromobenzoic acid, and four other organophosphates in human urine by solid phase extraction-high performance liquid chromatography-tandem mass spectrometry.* Analytical and bioanalytical chemistry, 2017. **409**(5): p. 1323-1332.

2. Food and Drug Administration, *Bioanalytical Method Validation - Guidance for Industry*. 2001.
